# Short-term incidence of protein-energy malnutrition is insufficient to evaluate long-term outcomes of hyposorptive bariatric surgery

**DOI:** 10.1007/s00464-024-10694-1

**Published:** 2024-01-29

**Authors:** Francesco Saverio Papadia, Gian Franco Adami, Giovanni Camerini

**Affiliations:** 1 Department of Surgical Sciences and Integrated Diagnostics (DISC), School of Medical and Pharmaceutical Sciences, University of Genoa, via Benedetto XV, nr. 6, 16132 Genoa, Italy; 2 Department of Internal Medicine (DIMI), School of Medical and Pharmaceutical Sciences, University of Genoa, via Benedetto XV, nr. 6, 16132 Genoa, Italy

The number of bariatric operations performed in the past 20 years has increased exponentially, due to a combination of increased prevalence of patients with obesity, greater acceptance of surgery as a therapeutic option by patients and stakeholders, and increased safety and effectiveness of bariatric surgical operations [1].

In their paper “The use of a total bowel length measurement protocol may reduce reoperations and complications after single-anastomosis duodenal switch”, Fair et al. compare retrospectively two groups of patients submitted to single-anastomosis duodenal switch with sleeve gastrectomy (SADI-S) [2]. In the experimental group, the authors adapt the common limb (CL) length to the total intestinal bowel length (“post-TBL group”). When compared with a control group with standard CL (250 cm for patients with diabetes, and 300 cm for patients without diabetes, “pre-TBL group”), post-TBL group patients received a longer CL length and showed a lower incidence of nutritional complications.

The main result of this study is precisely this: longer CL lengths lead to a lower incidence of protein-energy malnutrition (PEM) or diarrhea. Generally speaking, longer limb lengths lead also to lower long-term weight loss and maintenance.

We were struck by several points in this paper.

1. Pre-TBL group patients had a significantly lower preoperative albumin level when compared to the post-TBL group. This renders comparisons of postoperative albumin levels at 3 and 6 months difficult to interpret. Incidentally, albumin levels at 12 months were not significantly different.
2. Patients in the pre-TBL group were affected more frequently by diabetes mellitus, although the difference is not statistically significant. Patients with diabetes, especially if on insulin and decompensated, are more frail than non-diabetic patients, and experience a higher incidence of nutritional complications after hypoabsorptive surgery (3, 4). Patients’ overall frailty should be accounted for when evaluating complication rates.
3. We assume that patients in the pre-TBL group had a longer postoperative follow-up. Nonetheless, in absence of data on follow-up rate or length of follow-up, we fear the conclusions might not be generalizable. This highlights the importance of a standardized follow-up, especially for hypoabsorptive operations.
4. We believe that, in hypoabsorptive bariatric surgery, there is a learning curve also for postoperative nutritional patient management, apart from the purely surgical/technical one. We noticed that mean operative times in the post-TBL group were shorter than in the pre-TBL group, which is somehow surprising, considering that post-TBL patients necessitated measurement of the entire small bowel, which obviously adds to the total operative time. This shows that the authors acquired greater familiarity with SADI-S over time. It is possible that the different results were also due to better patient selection and nutritional management, in addition to a longer CL length.
5. The incidence of reoperation for protein malnutrition in the pre-TBL group appears to be extremely high (23% at a median follow-up of just 13 months). This fact reinforces the notion that pre-TBL patients might not be representative of the mature technique or follow-up.
6. With a relatively short follow-up, this study addresses the problem of early sporadic PEM, but not that of recurrent PEM, which represents an important clinical issue many years after the original hypoabsorptive bariatric surgery, responsible for significant long-term morbidity and mortality [3, 4]. Early sporadic PEM after hypoabsorptive bariatric surgery is usually due to insufficient early intestinal adaptation, or patient non-compliance [5]. We believe that the follow-up in this study is too short to show any differences in incidence of recurrent PEM, or of differences in weight loss, and therefore possibly inadequate to really estimate the safety of SADI-S.

In hypoabsorptive bariatric procedures, choosing the “right” limb lengths is difficult, and the choice is always a trade-off between the risk of nutritional complications versus long-term weight loss and maintenance, and remission of metabolic comorbidities. The most important message of this paper is that surgeons should measure the *entire* small bowel, when submitting patients to malabsorptive bariatric operations.

## References

[1] Clapp B, Ponce J, DeMaria E, Ghanem O, Hutter M, Kothari S, LaMasters T, Kurian M, English W (2022). American Society for Metabolic and Bariatric Surgery 2020 estimate of metabolic and bariatric procedures performed in the United States. Surg Obes Relat Dis.

[2] Fair L, Waddimba AC, Strothman P, Dwyer D, Anderton P, Bittle A, Ogola GO, Leeds S, Davis D (2023). The use of a total bowel length measurement protocol may reduce reoperations and complications after single-anastomosis duodenal switch. Surg Endosc.

[3] Papadia F, Carlini F, Longo G, Rubartelli A, Battistini M, Drago B, Adami GF, Marinari G, Camerini G (2023). Pyrrhic victory? Long-term results of biliopancreatic diversion on patients with type 2 diabetes and severe obesity. Surg Obes Relat Dis.

[4] Papadia FS, Carlini F, Rubartelli A, Battistini M, Cordera R, Adami GF, Camerini G (2022). Diabetes resolution at 10 years after biliopancreatic diversion in overweight and class 1 obese patients with type 2 diabetes. Obes Surg..

[5] Scopinaro N, Adami GF, Marinari GM, Gianetta E, Traverso E, Friedman D, Camerini G, Baschieri G, Simonelli A (1998). Biliopancreatic diversion. World J Surg.

